# Yttrium oxide nanoparticles induce selective cytotoxicity, genomic instability and ROS mitochondrial P53 mediated apoptosis in human pancreatic cancer cells

**DOI:** 10.1038/s41598-025-05088-9

**Published:** 2025-06-20

**Authors:** Hanan R. H. Mohamed, George M. Hakeem, Yasmin Abdel Latif, Shahd H. Elnawasani, Maria Nagy, Basma A. Mohamed, Rawan Essam, Gehan Safwat

**Affiliations:** 1 Department of Zoology, Faculty of Science, Cairo University, Giza, Egypt; 2 Faculty of Biotechnology, October University for Modern Sciences and Arts (MSA), 6th of October City, Egypt

**Keywords:** Y_2_O_3_-NPs, Cytotoxicity, Apoptosis, ROS, DNA damage, Mitochondrial integrity, Pancreatic cancer cancers, Genetics, Molecular biology, Biomarkers, Health care, Medical research

## Abstract

Pancreatic cancer is a hard-to-treat tumor with a poor prognosis. While traditional pancreatic cancer therapies can be effective, issues like cytotoxicity, low selectivity, and drug resistance still pose major challenges. Nanotechnology has shown promise in improving cancer diagnosis and treatment. Yttrium oxide nanoparticles (Y_2_O_3_-NPs), for example, have demonstrated potent selective cytotoxicity against triple negative breast cancer cells; but their effects on pancreatic cancer cells have not been explored. This study aimed to explore the impact of Y_2_O_3_-NPs on cell proliferation, DNA integrity, and oxidative stress in pancreatic cancer (PANC-1) and human skin fibroblast (HSF) cells. The cytotoxicity of Y_2_O_3_-NPs after 72 h were estimated using Sulforhodamine (SRB) cytotoxicity assay, while alkaline Comet assay was done to study genomic DNA integrity. Generation level of reactive oxygen species (ROS) and integrity of mitochondrial membrane potential were also analyzed. Apoptosis induction was investigated using Flow Cytometry and expression level of apoptotic (p53), anti-apoptotic (Bcl2) and mitochondrial (ND3) genes was measured using quantitative RTPCR. Our findings exhibited that Y_2_O_3_-NPs had strong selective cytotoxicity against PANC-1 cells with an IC50 value of 31.06 µg/ml, while having minimal effect on normal HSF cells (IC50 = 319.21 µg/ml). Treatment of PANC-1 cells with Y_2_O_3_-NPs at the IC50 concentration for 72 h significantly increased intracellular ROS levels and DNA damage, along with a notable reduction in mitochondrial membrane potential. Additionally, a significant rise in necrotic, early, and late apoptotic cells was observed, accompanied by downregulation of the anti-apoptotic Bcl2 gene and upregulation of the apoptotic p53 and mitochondrial ND3 genes. These findings highlight the selective toxicity of Y_2_O_3_-NPs towards cancerous PANC-1 cells, with minimal impact on normal cells. Y_2_O_3_-NPs appear to induce apoptosis in cancer cells by increasing ROS generation, damaging DNA, disrupting mitochondrial function, and triggering cell death. This study suggests that Y_2_O_3_-NPs may be a promising candidate for pancreatic cancer treatment. Further research is needed to fully explore their therapeutic potential.

## Introduction

Pancreatic cancer remains one of the most difficult malignancies to treat due to its aggressive progression and limited therapeutic options. Traditional treatment strategies, including surgery, chemotherapy, and radiation, have limited efficacy, especially in advanced stages where surgical resection is no longer viable^1,2^. Chemotherapy, with gemcitabine as a common first-line agent, works by inhibiting DNA synthesis and inducing apoptosis in pancreatic cancer cells^3^. Combination therapies, such as gemcitabine with nab-paclitaxel or FOLFIRINOX (folinic acid, fluorouracil, irinotecan, and oxaliplatin), have improved outcomes in some patient populations^4,5^.

Recent advances have introduced targeted therapies and immunotherapies as promising approaches for pancreatic cancer, aiming to enhance treatment efficacy while minimizing systemic toxicity. These therapies selectively inhibit critical pathways involved in cancer cell survival and proliferation. Specifically, they target molecules such as the epidermal growth factor receptor (EGFR), vascular endothelial growth factor (VEGF), programmed cell death protein 1 (PD-1), cytotoxic T-lymphocyte-associated protein 4 (CTLA-4), and poly (ADP-ribose) polymerase (PARP), which play key roles in pancreatic cancer progression^6–8^.

Despite these advancements, pancreatic cancer’s prognosis remains poor, highlighting the ongoing need for novel therapeutic strategies and personalized treatments tailored to the molecular profiles of individual tumors^2^. Nanotherapy, which involves the use of nanoparticles for diagnosis, targeted drug delivery, and therapeutic intervention, has emerged as a promising field. Nanoparticles, ranging from 1 to 100 nm, offer unique benefits, such as enhanced stability, prolonged circulation times, and the ability to penetrate biological barriers. These properties make nanoparticles ideal candidates for selectively targeting diseased tissues while minimizing systemic toxicity^9,10^.

Nanotherapy has overcome several challenges associated with traditional chemotherapy, such as poor drug solubility and non-specific distribution^11,12^. Yttrium oxide nanoparticles (Y_2_O_3_-NPs) have gained significant attention in biomedical research due to their unique physicochemical properties, which contribute to both antioxidant capabilities and therapeutic potential. These nanoparticles can scavenge reactive oxygen species (ROS), mitigating oxidative stress-induced damage, a key factor in diseases like cancer and neurodegenerative disorders^13,14^. Y_2_O_3_-NPs have also demonstrated promise in targeted drug delivery due to their biocompatibility and surface chemistry, enabling the precise delivery of therapeutic agents and minimizing off-target effects^13^. Furthermore, they can be functionalized with biomolecules, such as antibodies or drugs, to increase their specificity and therapeutic efficacy^14,16^.

In cancer therapy, Y_2_O_3_-NPs have shown cytotoxic effects against cancer cells by inducing apoptosis, disrupting cellular signaling pathways, and altering the tumor microenvironment^17,18^. They also offer potential for combination therapies, such as co-delivery of chemotherapeutic agents or synergistic use with other nanoparticles, enhancing overall treatment outcomes^17,19^. Recent studies demonstrated that Y_2_O_3_-NPs exhibit selective potent cytotoxicity and genotoxicity against human triple-negative breast cancer cells (MDA-MB-231) by inducing excessive ROS generation, DNA damage, apoptosis, and ferroptosis^18^. Conversely, Y_2_O_3_-NPs were found to be safe and non-genotoxic in human normal retinal RPE1 and dermal fibroblast HDF cells, although they caused significant cytotoxicity in human embryonic kidney (HEK293) cells, leading to ROS generation, DNA damage, mitochondrial membrane disruption, and apoptosis^20^.

The differential effects of Y_2_O_3_-NPs on cancer cells versus normal cells raise important questions about their potential in cancer therapy. Their selective cytotoxicity in triple-negative breast cancer cells holds promise for more effective and less toxic cancer treatments^18^. However, the observed toxicity in HEK293 cells emphasizes the need for further research into the safety and mechanisms of Y_2_O_3_-NPs, especially regarding off-target effects on healthy tissues^20^. Understanding these mechanisms is crucial to developing safe and effective cancer therapies. Despite the growing body of research on Y_2_O_3_-NPs, there is a lack of data regarding their impact on human pancreatic cancer cells. This gap prompted the current study, which investigates the effects of Y_2_O_3_-NPs on the viability of human normal skin fibroblasts (HSF) and pancreatic cancer (PANC-1) cells. Specifically, we assessed genomic and mitochondrial DNA integrity, ROS generation, and apoptosis induction in PANC-1 cells. Cell viability was measured using the Sulforhodamine B (SRB) assay, while genomic DNA integrity was evaluated through the alkaline Comet assay. ROS generation and mitochondrial membrane potential were analyzed using 2,7-Dichlorofluorescein diacetate and Rhodamine 123, respectively. Apoptosis was assessed by Flow Cytometry, and the expression of apoptotic (p53), anti-apoptotic (Bcl2) and mitochondrial NADH dehydrogenase 3 (ND3) genes was quantified using quantitative RT-PCR (qRT-PCR).

## Materials and methods

### Chemicals

The Y_2_O_3_-NPs used in this study were purchased from Sigma-Aldrich Company (St. Louis, MO, USA) in the form of white powders with a product number of 544892. The particles’ size was less than 50 nm, and they had a purity of 99.9% trace metals. All other materials and supplies utilized in this study were sourced at the high molecular grade level to ensure consistency and accuracy in the experimental procedures.

### Characterization of Y_2_O_3_-NPs

Characterization of Y_2_O_3_-NPs was carried out as per the methodology described by Emad et al.^18^. The purity of purchased Y_2_O_3_-NPs powders was confirmed using X-ray diffraction analysis (XRD). Dynamic laser scattering (DLS) also assessed the stability and well distribution of suspended Y_2_O_3_-NPs with a Zeta Potential of − 53.2 mV, and polydispersity index value of 0.630. Transmission electron microscope (TEM) imaging was conducted to examine the morphology of Y_2_O_3_-NPs, revealing a spherical and cubic shape with an average particles’ size of 14.00 nm^18^.

### Cell culture

Human Pancreatic cancer (PANC-1) and normal skin fibroblast (HSF) cell lines were obtained from Nawah Scientific Inc. (Mokatam, Cairo, Egypt). The cells were cultured in DMEM medium supplemented with 10% inactivated fetal bovine serum, 100 units/mL penicillin, and 100 mg/mL streptomycin. The cultures were maintained in an incubator at 37 °C with 5% CO_2_.

### Cytotoxicity estimation using SRB colorimetric assay

Sulforhodamine B (SRB) assay was done to assess effect of Y_2_O_3_-NPs on viability of human pancreatic cancer PANC-1 and normal HSF cells as previously described^21,22^. Pancreatic cancer PANC-1 and normal HSF cell suspensions (100 µL) were separately cultured in 96-well plates and incubated for 24 h in complete media. Subsequently, the cells were treated with varying concentrations of Y_2_O_3_-NPs (0.2, 2, 20, 200 and 2000 µg/mL for 72-h. Following exposure to nanoparticles, the cultured cells were fixed, washed with distilled water, and incubated with SRB solution (0.4% w/v) for 10 min at room temperature in the dark. Plates were then washed with acetic acid (1%) and allowed to dry overnight. The protein-bound SRB stain was dissolved, and the absorbance was measured at 540 nm using a BMG LABTECH®-FLUO Star Omega microplate reader (Ortenberg, Germany). GraphPad Prism software was employed to determine the IC50 of the three treated replicates.

### Cell treatment

Pancreatic cancer PANC-1 cells were cultured in T25 flasks under appropriate conditions. The cultured cells were then divided into treated and control cells. Control cells were treated with DMSO at a concentration less than 0.1%, while treated cells were exposed to Y_2_O_3_-NPs at a concentration equal to the IC50 value. After 72 h post-treatment, all PANC-1 cells underwent centrifugation and trypsinization for cell harvesting. The harvested PANC-1 cells were washed twice with ice-cold PBS and stored at − 80 °C in PBS for further molecular analysis. Triplicate was done for the tested concentration of Y_2_O_3_-NPs to ensure accuracy and consistency in the results.

### Screening the intracellular ROS generation using DCFH-DA

To assess the impact of Y_2_O_3_-NPs treatment on ROS production within PANC-1 cells, 2,7-dichlorofluorescin diacetate (DCFH-DA) dye was utilized following the protocol outlined by Siddiqui et al.^23^. Briefly, PANC-1 cell suspension cells were gently mixed with DCFH-DA dye at a concentration of 20 mM and incubated for 30 min in the dark at room temperature. The dye slowly penetrated into the cells and specifically reacted with ROS to produce dichlorofluorescein (DCF), a fluorescent molecule. Following the incubation period, the mixture of dye and cells was spread onto a clean slide and examined using an epi-fluorescent microscope at 200× magnification. Images were captured to visualize the fluorescent signal indicative of ROS production within the PANC-1 cells.

### Measuring DNA damage level using alkaline Comet assay

An alkaline comet assay was conducted to assess the level of DNA damage in both Y_2_O_3_-NPs-treated and untreated PANC-1 cells^24,25^. A 15 µL cell suspension was mixed with 60 µL of low melting agarose (0.5%) and spread onto a pre-coated slide with normal melting agarose (1%). The gel was allowed to solidify, followed by incubation in a cold lysis buffer containing DMSO and Triton-X100 for 24 h at 4 °C in the dark. Subsequently, the slides were exposed to freshly prepared alkaline electrophoresis buffer (pH > 12) for 15 min and electrophoresed at 25 V and 300 mA for 30 min. Post-electrophoresis, the slides were neutralized, fixed, dried, and stained with ethidium bromide for imaging. The captured Comet nuclei with varying levels of DNA damage were analyzed using COMETSCORE (TM) software. The measured Comet parameters, including tail length, %DNA in tail, and tail moment, were expressed as mean ± standard deviation (SD).

### Assessing the mitochondrial membrane potential integrity

The impact of Y_2_O_3_-NPs on the integrity of mitochondrial membrane potential was investigated utilizing a modified protocol outlined by Zhang et al.^26^ with slight changes. Cell suspensions of both untreated and Y_2_O_3_-NPs-treated PANC-1 cells were each mixed with 10 mg/ml of Rhodamine-123 fluorescent dye and then incubated for 1 h at 37 °C in the absence of light. Subsequently, the cells were washed twice with PBS and then placed on a clean sterile slide. Fluorescence emitted from Rhodamine-123 was visualized and captured using an epi-fluorescence microscope at 200× magnification. This allowed for the assessment of any changes in membrane potential integrity due to exposure to Y_2_O_3_-NPs.

### Apoptosis detection using flow cytometry

Flow cytometric analysis was conducted to assess apoptosis and necrosis induction in untreated and Y_2_O_3_-NPs-treated PANC-1 cells. Annexin V- Fluorescein isothiocyanate (FITC) apoptosis detection kit (Abcam Inc., Cambridge Science Park Cambridge, UK) coupled with two fluorescent channels Flow Cytometry were used to discriminate between apoptotic and necrotic cell populations. After 72 h of exposure to Y_2_O_3_-NPs, PANC-1 cells were harvested by trypsinization and washed twice with ice-cold PBS (pH 7.4). The harvested cells were incubated in the dark with Annexin V-FITC/propidium iodide (PI) solution for 30 min at room temperature. Subsequently, they were analyzed using the ACEA Novocyte flow cytometer (ACEA Biosciences Inc., San Diego, CA, USA), with FITC and PI fluorescent signals detected by FL1 and FL2 signal detectors, respectively (λex/em 488/530 nm for FITC and λex/em 535/617 nm for PI). Each sample was subjected to acquisition of 12,000 events, and positive FITC and/or PI cells were quantified using quadrant analysis and calculated with ACEA NovoExpress software (ACEA Biosciences Inc., San Diego, CA, USA).

### Measuring mRNA expression level of p53, Bcl2 and ND3 genes

Quantitative Real-Time PCR (qRT-PCR) was performed to investigate how exposure of PANC-1 cells to Y_2_O_3_-NPs influences the mRNA expression level of p53 and Bcl2 apoptotic genes and the mitochondrial gene ND3. Total RNA was extracted from cultured PANC-1 cells using Thermo Fisher Scientific’s GeneJET RNA Purification Kit (USA). Subsequently, 1 µg of extracted RNA was used for cDNA synthesis with the cDNA Reverse Transcription Kit from Applied Biosystems (Foster City, CA, USA). The mRNA expression levels of p53, Bcl2, and ND3 genes were quantified using the StepOnePlus Real-Time PCR System (Applied Biosystems). For amplification, qRT-PCR was conducted with SYBER Green PCR Master Mix (Applied Biosystems, USA) and specific primers^27–29^ as listed in Table 1. The expression level of the amplified genes was measured using the comparative Ct (ΔΔCt) method, with GAPDH serving as the housekeeping gene. Results were reported as mean ± SD.

**Table 1:** Sequences of primers used in qRT-PCR.

Gene	Strand	Primer’s sequences
GAPDH	Forward	5′-GAAGGTGAAGGTCGGAGTCA-3′
	Reverse	5′-GAAGATGGTGATGGGATTTC-3′
ND3	Forward	5′-CGCCGCCTGATACTGGCAT-3′
	Reverse	5′-CTAGTATTCCTAGAAGTGAG-3′
BCL-2	Forward	5′-TCCGATCAGGAAGGCTAGAGT-3′
	Reverse	5′-TCGGTCTCCTAAAAGCAGGC-3′
P53	Forward	5′-CAGCCAAGTCTGTGACTTGCACGTAC-3′
	Reverse	5′-CTATGTCGAAAAGTGTTTCTGTCATC-3′

### Statistical analysis

In this study the obtained results were analyzed using the Statistical Package for the Social Sciences (SPSS) and are presented as mean ± SD. A comparison between treated and untreated cells was conducted using the unpaired Student’s t-test at *p* < 0.05.

## Results

### Y_2_O_3_-NPs is selectively cytotoxic against PANC-1 cells

**Fig. 1:**
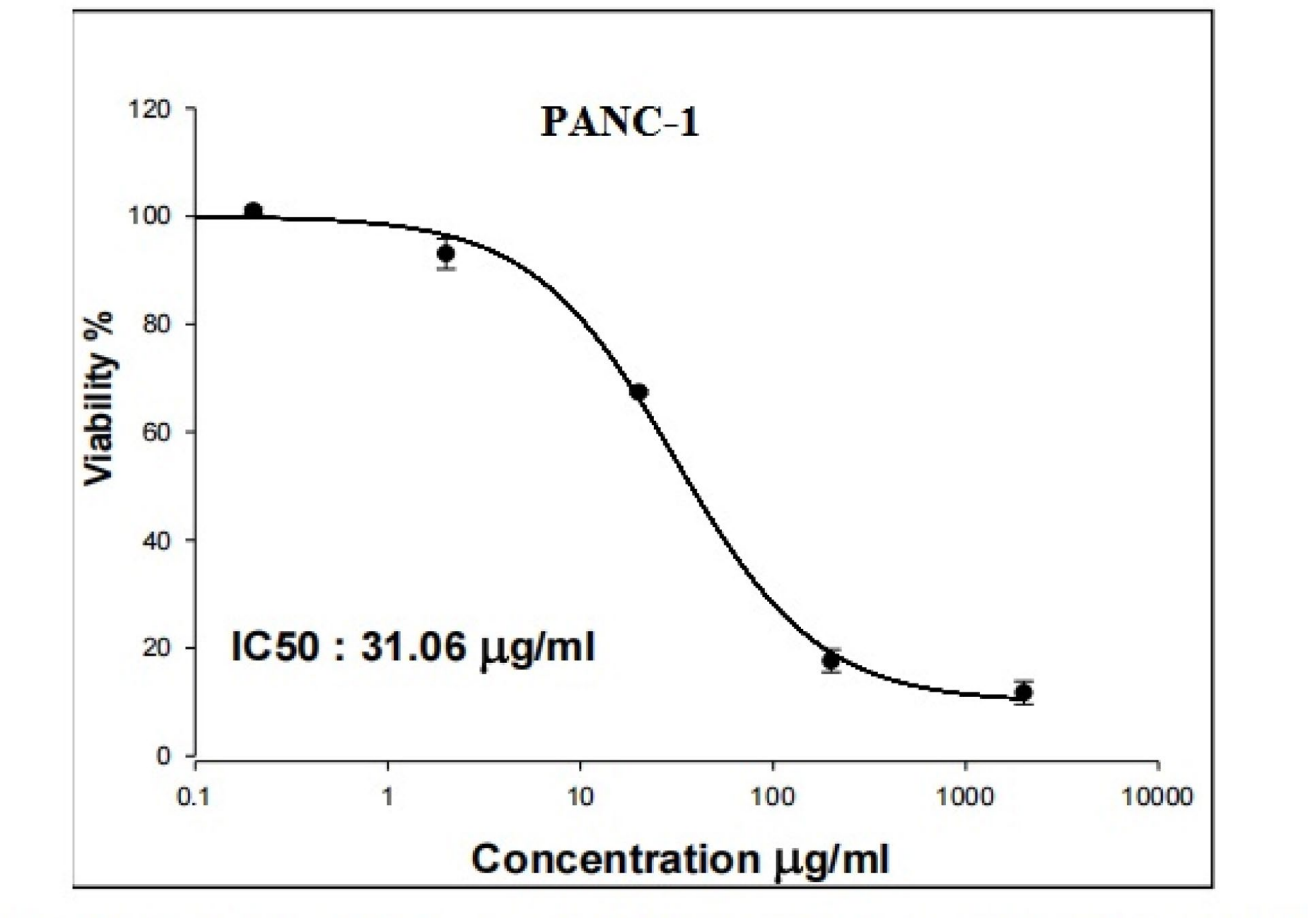
Viability of pancreatic PANC-1 cancer cells after exposure to five concentrations of Y_2_O_3_-NPs for 72 h.

**Fig. 2:**
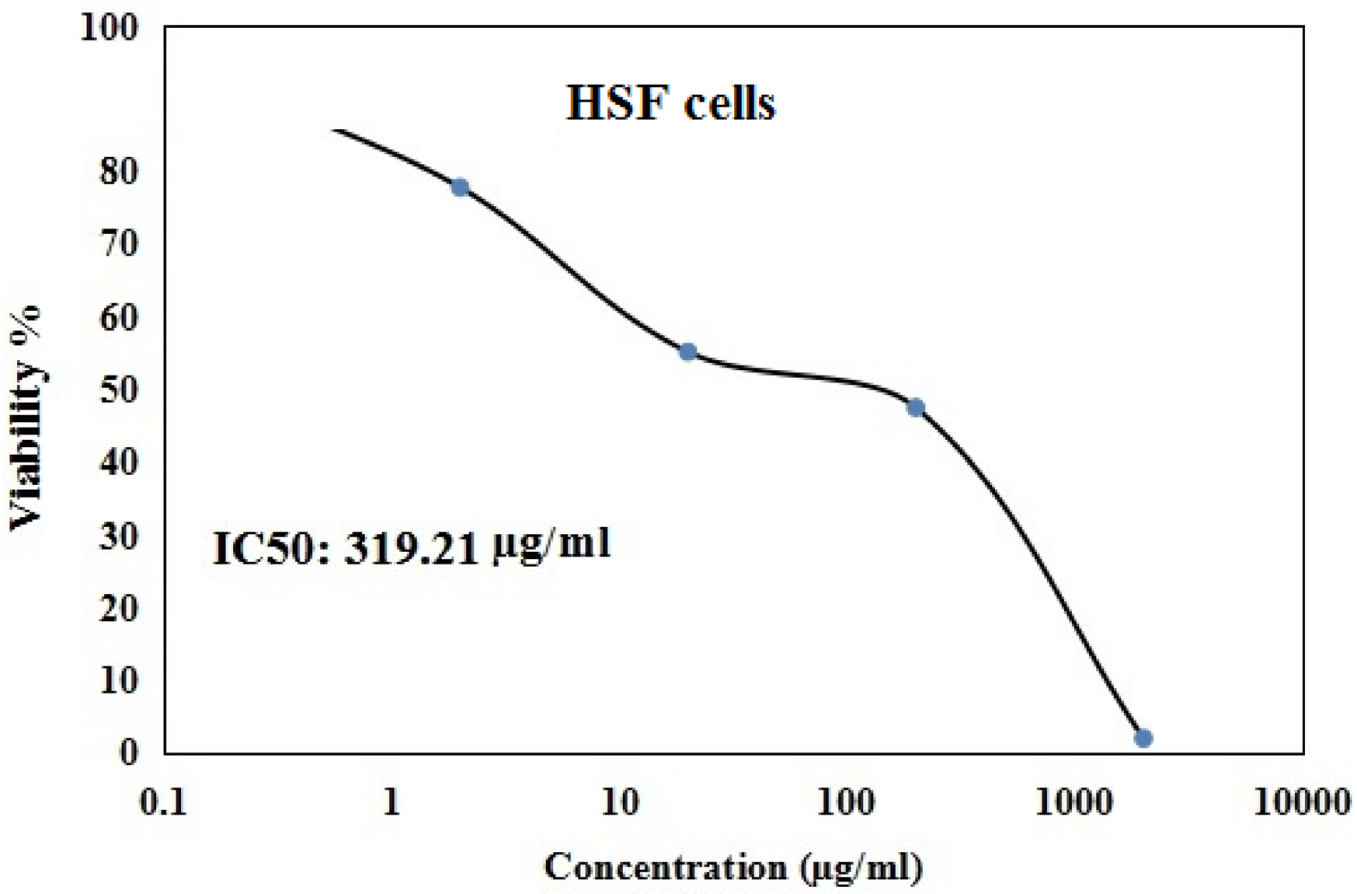
Viability of normal HSF cells after exposure to five concentrations of Y_2_O_3_-NPs for 72 h.

As displayed in Figs. 1 and 2 Y_2_O_3_-NPs exhibited a remarkable selective proliferation inhibition and death induction in aggressive pancreatic PANC-1 cancer cells, as evidenced by a high concentration-dependent reduction noticed in PANC-1 cell viability with an IC50 value of 31.06 µg/ml (Fig. 1) compared to the slight decrease observed in normal HSF cell viability with IC50 value of 319.21 µg/ml (Fig. 2) after cell exposure to various concentrations of Y_2_O_3_-NPs (0.2, 2, 20, 200 or 2000 µg/ml) for 72 h. The high selectivity index value of 10.27 further underscores the preferential targeting of PANC-1 cells by Y_2_O_3_-NPs.

### Y_2_O_3_-NPs over-generate ROS in PANC-1 cells

**Fig. 3:**
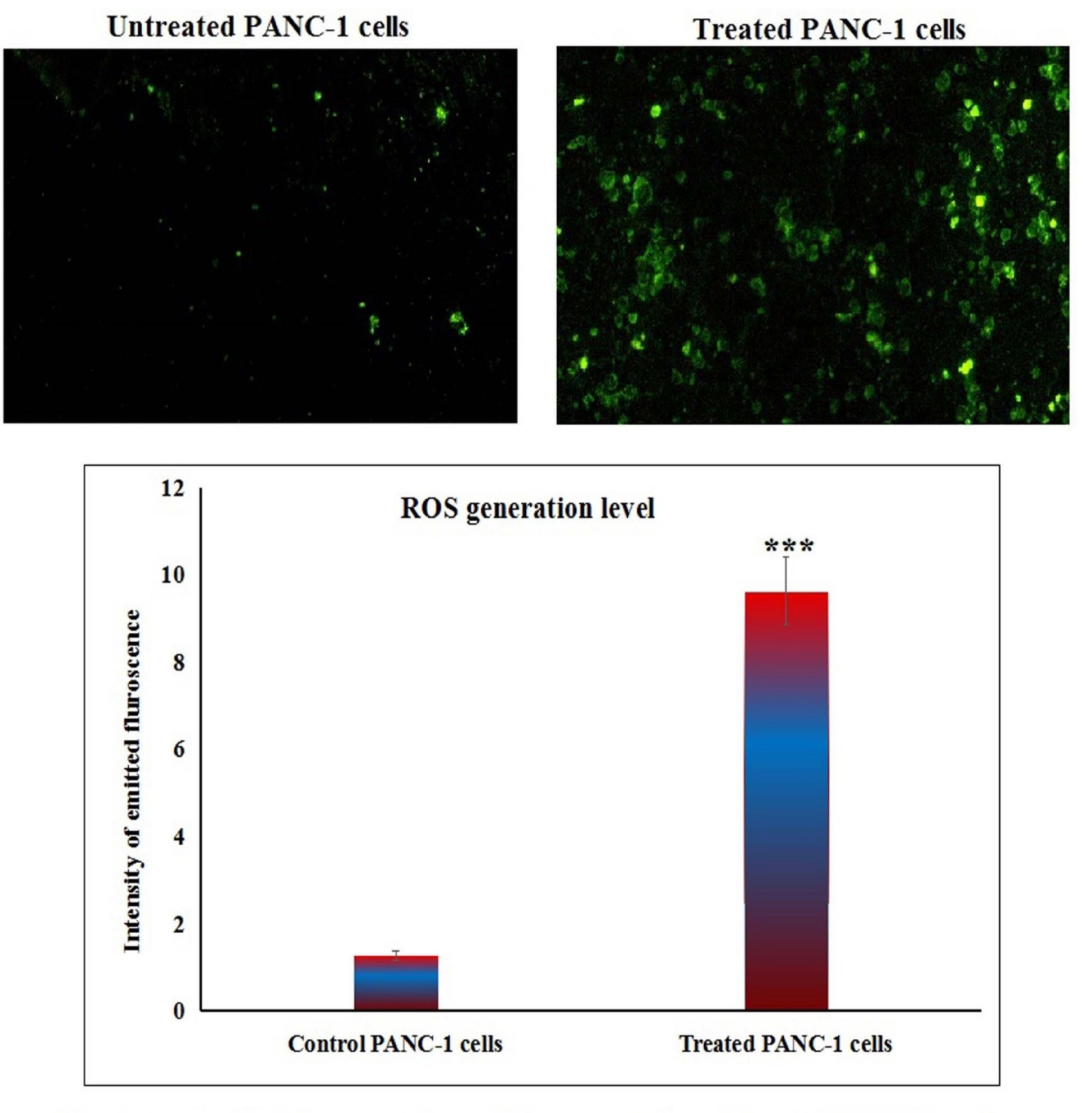
Level of ROS generation within untreated and treated PANC-1 cells with an IC50 concentration of Y_2_O_3_-NPs for 72 h.

Measuring the ROS generation with PANC-1 cells using 2,7-DCFH-DA dye demonstrated a notable increase (*p* < 0.001) in ROS production within pancreatic PANC-1 cancer cells after a 72-h treatment with Y_2_O_3_-NPs. This remarkable increase was shown in Fig. 3 through the considerable increase observed in fluorescent light intensity emitted by Y_2_O_3_-NPs treated PANC-1 cells compared to that emitted by untreated PANC-1 cells.

### Y_2_O_3_-NPs destroy genomic DNA in PANC-1 cells

**Fig. 4:**
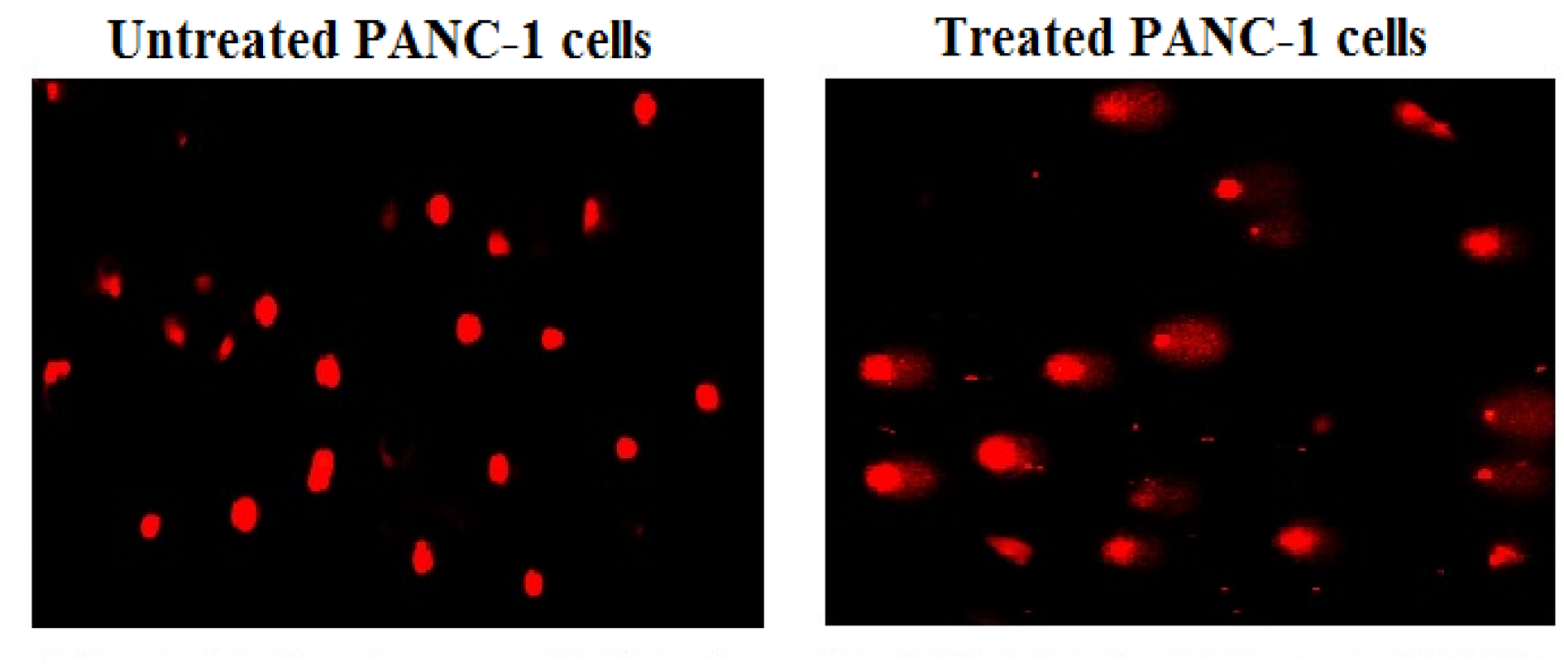
Examples for the scored Comet nuclei with intact and various DNA damage in the untreated and treated PANC-1 cells with an IC50 concentration of Y_2_O_3_-NPs for 72 h.

The results of the alkaline Comet assay demonstrated that exposure to Y_2_O_3_-NPs for 72 h dramatically damaged the genomic DNA integrity of pancreatic PANC-1 cancer cells. This effect was manifested by the statistically significant (*p* < 0.01) increases noticed in tail length, %DNA in the tail, and tail moment in PANC-1 cells after treatment with an IC50 concentration (31.06 µg/ml) of Y_2_O_3_-NPs when compared to untreated control PANC-1 cells, as exhibited in Table 2 and Fig. 4.

**Table 2:** Integrity of genomic DNA in untreated and Y_2_O_3_-NPs treated PANC-1 cells.

		Tail length (px)	%DNA in tail	Tail moment
PANC-1 cells	Untreated	6.87 ± 0.73	35.82 ± 2.88	2.48 ± 0.45
	Treated	13.13 ± 1.17 **	49.68 ± 2.94 **	6.55 ± 0.65**

Results are expressed as mean ± SD.

**, ***Indicates statistical significant difference from the compared untreated control cells at *p* < 0.01 and 0.001, respectively using *independent student t-test.*

### Y_2_O_3_-NPs disrupt the mitochondrial membrane potential integrity in PANC-1 cells

**Fig. 5:**
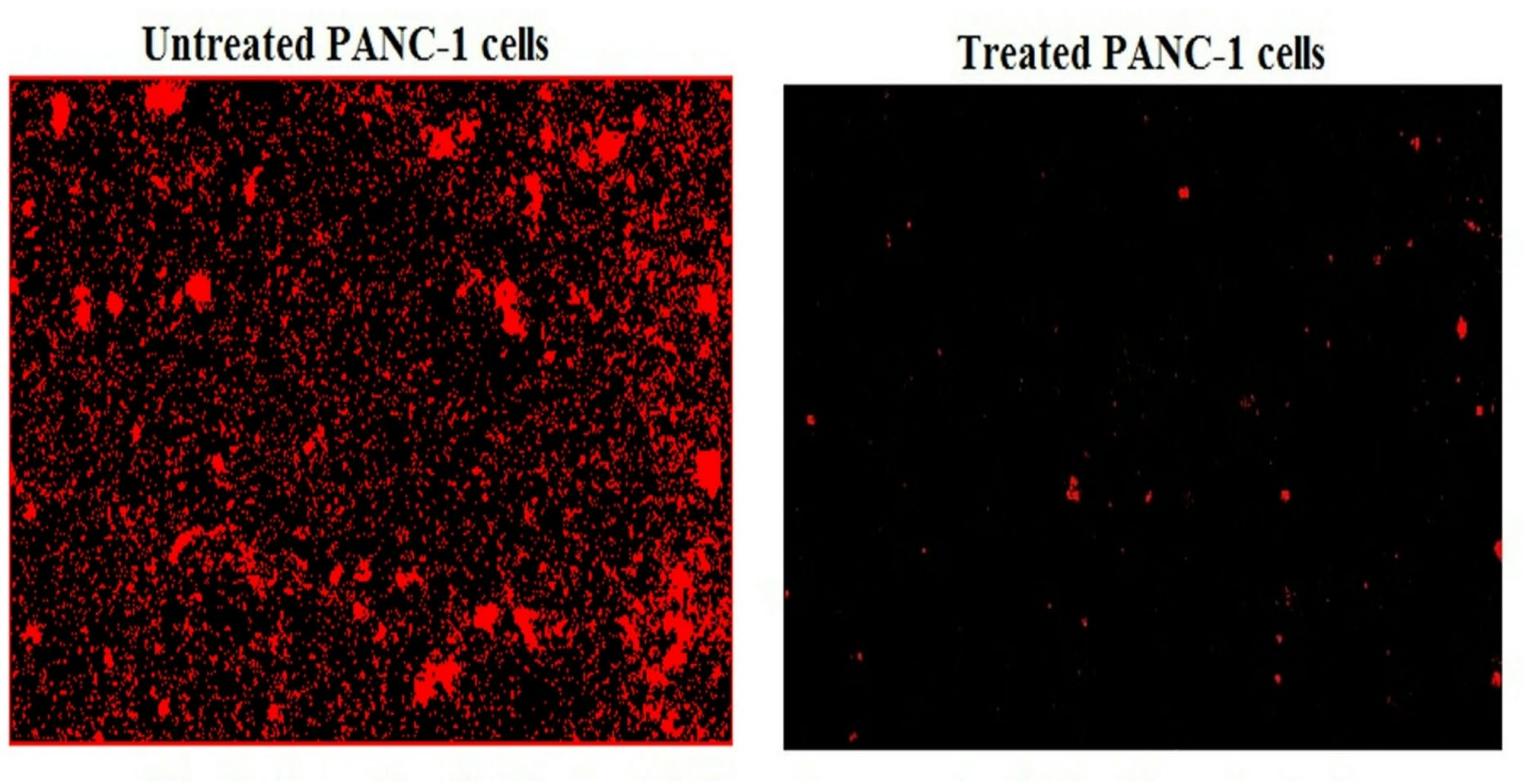
Integrity of mitochondrial membrane potential in the untreated and treated PANC-1 cells with an IC50 concentration of Y_2_O_3_-NPs for 72 h.

Upon treatment with Y_2_O_3_-NPs at a concentration of IC50 value (31.06 µg/ml) for 72 h, a high reduction in the integrity of mitochondrial membrane potential was noticed in Y_2_O_3_-NPs treated PANC-1 cells as opposed to those that were not treated with Y_2_O_3_-NPs. As depicted in Fig. 5 the marked decrease in mitochondrial membrane potential integrity was demonstrated by a notable decrease in fluorescence light intensity emitted by Y_2_O_3_-NPs-treated PANC-1 cells compared to that highly emitted by untreated PANC-1 cells.

### Y_2_O_3_-NPs induce apoptosis and necrosis of PANC-1 cells

**Fig. 6:**
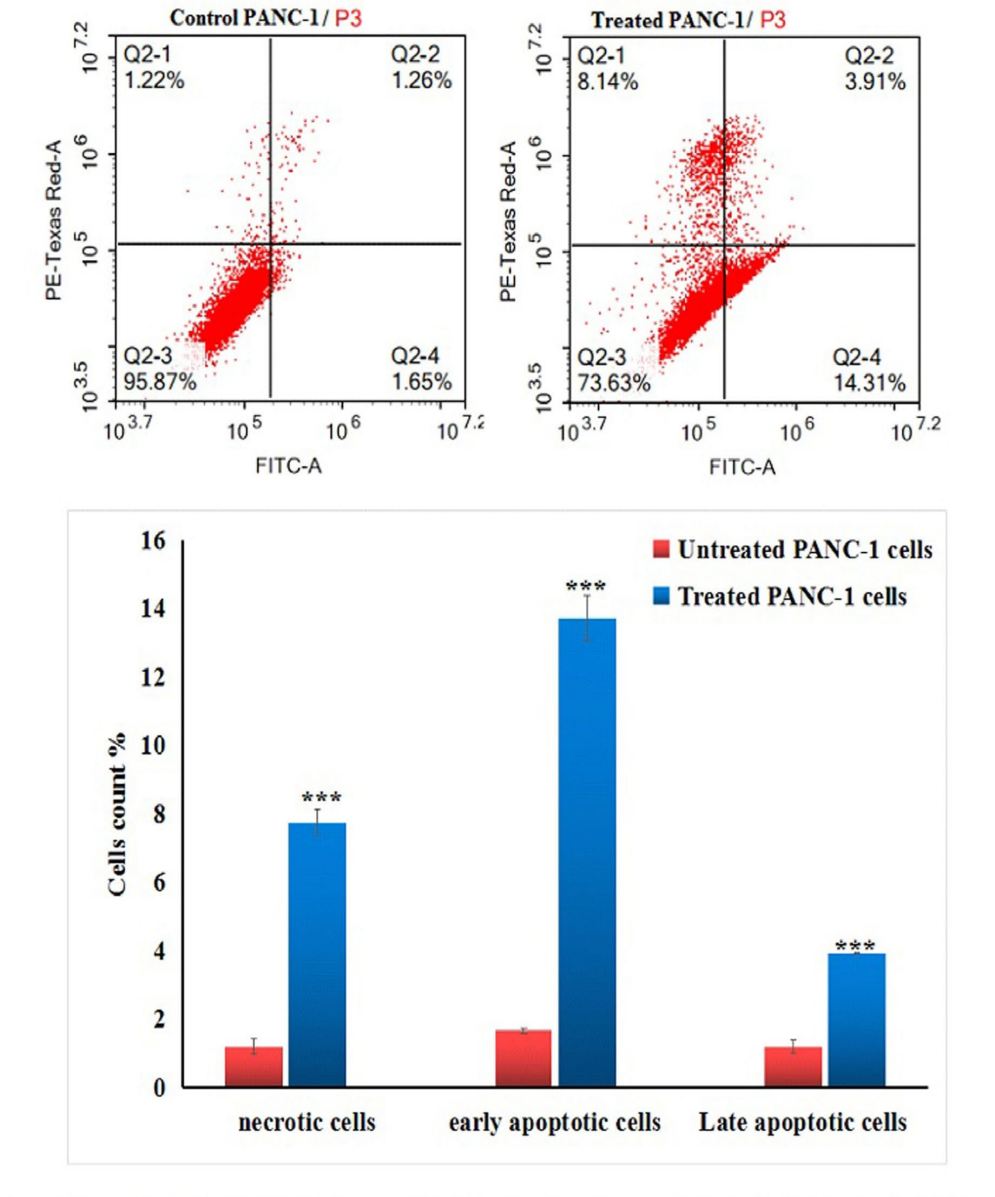
Apoptosis induction in the untreated (control) and treated PANC-1 cells with an IC50 concentration of Y_2_O_3_-NPs for 72 h. Q2-1 denotes necrosis phase; Q2-2 denotes late apoptosis phase, Q2-3 denotes normal viable cells and Q2-4 denotes early apoptosis phase.

Flow cytometric analysis revealed that treatment of pancreatic PANC-1 cancer cells with an IC50 concentration (31.06 µg/ml) of Y_2_O_3_-NPs resulted in a significant stimulation of apoptosis and necrosis. This potent cytotoxic effect is illustrated in Fig. 6 through the statistically significant (*P* < 0.001) elevations in the number of early and late apoptotic, as well as necrotic PANC-1 cells observed 72 h after Y_2_O_3_-NPs treatment compared to the untreated PANC-1 cells.

### Y_2_O_3_-NPs dysregulate apoptotic and mitochondrial genes expression in PANC-1 cells

The data of qRT-PCR demonstrated that PANC-1 cells treatment with Y_2_O_3_-NPs at a concentration of IC50 value (31.06 µg/ml) statistically significantly up-regulated the mRNA expression level of apoptotic p53 and mitochondrial ND3 genes compared to their expression level in the untreated PANC-1 cells (Table 3). In contrast, the expression level of the anti-apoptotic Bcl2 gene was statistically significantly down regulated after PANC-1 cells exposure to Y_2_O_3_-NPs for 72 h as shown in Table 3.

**Table 3:** The expression level of p53, ND3 and Bcl2 genes in untreated and Y_2_O_3_-NPs treated PANC-1 cells.

		p53	ND3	Bcl2
PANC-1 cells	Untreated	1.00 ± 0.00	1.00 ± 0.00	1.00 ± 0.00
	Treated	2.67 ± 0.47**	7.30 ± 0.35***	0.14 ± 0.05***

Results are expressed as mean ± SD.

**, ***Indicates statistical significant difference from the compared untreated control cells at *p* < 0.01 and 0.001, respectively using *independent student t-test.*

## Discussion

Pancreatic cancer is known for its high degree of invasiveness and resistance to conventional chemotherapeutic drugs, making it a challenging disease to treat effectively. The use of nanomedicine has emerged as a promising approach to combat these challenges, with the ability to enhance treatment outcomes while minimizing adverse reactions. Previous research has shown the selective cytotoxicity and genotoxicity of Y_2_O_3_-NPs towards triple negative MDA-MB-231 cells, indicating their potential effectiveness in targeting cancer cells^18^. However, there is a lack of information on the effects of Y_2_O_3_-NPs on pancreatic cancer cells. Therefore, this study was conducted to address this gap in knowledge and shef light on the potential utility of Y_2_O_3_-NPs in the treatment of pancreatic cancer.

The findings of this study demonstrated the promising selective cytotoxic effects of Y_2_O_3_-NPs on pancreatic cancer cells with negligible toxicity on normal HSF cells, highlighting their potential as a novel therapeutic approach for this challenging disease. This targeted cytotoxicity was manifested through a marked concentration-dependent reduction noticed in viability of PANC-1 cells after 72 h of Y_2_O_3_-NPs treatment, in contrast to the slight decrease observed in the viability of Y_2_O_3_-NPs-treated normal HSF cells consistently with recent findings that demonstrated the potent selective cytotoxicity of Y_2_O_3_-NPs towards triple negative MDA-MB-231 breast cancer cells, while being safe for human normal retinal RPE1 and dermal fibroblasts HDF cells^18^. Similarly, exposure to different concentrations of Y_2_O_3_-NPs for 24 and 48 h caused a significant decrease in the viability of U87MG malignant glinoma and PC3 prostate cancer cells as at a concentration and time dependent manner^30^.

The behavior of Y_2_O_3_-NPs upon incubation with cells plays a pivotal role in understanding their biological effects and underlying mechanisms of cytotoxicity. The stability of Y_2_O_3_-NPs in the culture medium was confirmed through DLS and TEM analysis, as reported by Emad et al.^18^, which demonstrated that the Y_2_O_3_-NPs maintain their spherical shape and remain well-dispersed. This stability is essential for ensuring their effective cellular uptake. Upon exposure to cells for 72 h, Y_2_O_3_-NPs are internalized within cells^31^, and the Y_2_O_3_-NPs cytotoxicity observed in this study is primarily attributed to the Y_2_O_3_-NPs themselves, rather than any released drugs or ions. The internalization of Y_2_O_3_-NPs likely triggers various cellular responses, including oxidative stress, DNA damage, and mitochondrial dysfunction, all of which are known to be associated with nanoparticle-induced toxicity^18,31^.

Consequently, to fully understand the mechanisms underlying the potent cytotoxic effects of Y_2_O_3_-NPs on pancreatic cancer cells, DNA integrity, ROS generation level, mitochondrial membrane potential integrity and apoptosis induction were assessed in PANC-1 cells. Screening the genomic DNA integrity using alkaline Comet assay demonstrated dramatic genomic DNA damage induction by Y_2_O_3_-NPs in PANC-1 cells through reported significant elevations reported in tail length, %DNA in tail and tail moment measured in Y_2_O_3_-NPs-treated PANC-1 cells versus untreated PANC-1 cells. These results are in consistent with the recent finding that DNA damage is markedly elevated by Y_2_O_3_-NPs in triple negative MDA-MB-231 breast cancer cells^18^.

This detectable high DNA damage induction in Y_2_O_3_-NPs treated PANC-1 cells is anticipated due to the high production of ROS demonstrated in the aggressive PANC-1 cancer cells through high elevations in the intensity of the emitted fluorescent light by Y_2_O_3_-NPs treated PANC-1 cells versus untreated cells. Intensive production of the highly reactive ROS destabilizes the equilibrium between antioxidants and oxidants and also damage cellular macromolecules including DNA causing various DNA lesions such as oxidized based and DNA breaks^32,33^. Induction of DNA breaks after PANC-1 cells exposure to Y_2_O_3_-NPs was manifested by the remarkable elevations in measured alkaline Comet assay parameters since alkaline Comet assay sensitively and accurately detects both single and double stranded DNA breaks in each cell separately^24^. These consequences parallel with those of recent studies that found that exposure of aggressive triple negative MDA-MB-231 breast and melanoma A-375 cancer cells to Y_2_O_3_-NPs increased ROS formation, which disrupted cellular homeostasis, triggered oxidative stress and induced cell death^17,18^. The induction of oxidative stress was evidenced by a statistically significant increase in malondialdehyde levels in the aggressive triple-negative MDA-MB-231 breast cancer cells following exposure to Y_2_O_3_-NPs^18^.

Overproduction of ROS also attack and damages mitochondria a long with inducing DNA breaks^34^. Mitochondrial dysfunction in Y_2_O_3_-NPs treated PANC-1 cells was depicted by the remarkable reduction in the integrity of mitochondrial membrane potential noticed after PANC-1 cells exposure to Y_2_O_3_-NPs. This finding corresponds to a recent study exhibited that treatment of HEK293 cells with Y_2_O_3_-NPs damages mitochondria and increases mitochondrial membrane permeability^20^.

The excessive production of ROS creates a stressful environment for the mitochondria, disrupting the permeability of its membrane and affecting its overall biophysical properties. As a result, the biochemical functions of various transporters and respiratory enzymes in both the inner and outer mitochondrial membranes are compromised. Moreover, the depolarization of mitochondria results in the release of important solutes such as NAD+ and NADH into the cytosol, further exacerbating cellular dysfunction. This cascade of events ultimately forces the cell into apoptosis, highlighting the critical role of ROS in triggering mitochondrial stress and subsequent cell death^35^. Likewise, DNA damage lesions, specifically DNA breaks, pose a significant threat to the genomic DNA integrity of cells. A single DNA break has the potential to be fatal, as it can lead to disruption of crucial genetic information and ultimately result in cell death through apoptosis. This type of DNA damage is particularly dangerous due to its ability to irreversibly harm the cell and impair its functionality^36^.

Excessive generation of ROS such as superoxide anions, hydrogen peroxide, and hydroxyl radicals, directly damages the cellular components, including DNA and mitochondria leading to oxidative stress. This oxidative stress activates several cellular pathways, including the DNA damage response and apoptotic signaling cascades. The subsequent mitochondrial dysfunction, characterized by loss of membrane potential, cytochrome c release, and caspase activation, leads to apoptosis and cell death. These mechanisms are crucial for the selective toxicity of Y_2_O_3_-NPs against cancer cells, highlighting their potential for use in cancer therapy^18–20^. Consequently, our findings of Y_2_O_3_-NPs induced excessive ROS generation, marked DNA breaks and disruption of mitochondrial membrane permeability, forcing PANC-1 cancer cells to die through apoptosis. Apoptosis of Y_2_O_3_-NPs treated PANC-1 cells was demonstrated by the significant elevations observed in the number of apoptotic (early and late) and necrotic PANC-1 cells after exposure to Y_2_O_3_-NPs versus untreated PANC-1 cells. Recent studies have consistently proved significant elevations in the number of early and late apoptotic phases of triple negative MDA-MB-231 breast cancer and HEK293 cells after exposure to Y_2_O_3_-NPs^18,20^.

Our findings show that Y_2_O_3_-NPs induce excessive ROS generation, significant DNA breaks, and disruption of mitochondrial membrane permeability, driving PANC-1 cancer cells to undergo apoptosis. This was evidenced by a marked increase in both early and late apoptotic as well as necrotic PANC-1 cells following Y_2_O_3_-NPs exposure compared to untreated cells. Recent studies have similarly demonstrated significant elevations in the number of early and late apoptotic cells in triple-negative MDA-MB-231 breast cancer and HEK293 cells after exposure to Y_2_O_3_-NPs^18,20^.

Apoptosis, or programmed cell death, is a vital process crucial for maintaining tissue homeostasis and eliminating potentially harmful cells from the body. Several key molecular players play significant roles in modulating apoptosis, for example the interplay between tumor suppressor p53, mitochondrial ND3, and anti-apoptotic Bcl2 genes underscores the complexity of apoptosis regulation. Upregulation of the apoptotic p53 gene activates apoptosis through the direct induction of pro-apoptotic factors and the indirect downregulation of anti-apoptotic genes like anti-apoptotic Bcl2 gene. In addition, the mitochondrial ND3 gene, which encodes a subunit of mitochondrial respiratory chain complex I, functions as an apoptosis inducing factor through its ability to release pro-apoptotic factors^37,38^. Our qRT-PCR findings revealed that Y_2_O_3_-NPs induce apoptosis in PANC-1 cells through the significant upregulation of the expression level of apoptotic p53 and ND3 genes simultaneously with remarkable down regulation of the anti-apoptotic Bcl2 gene expression level noticed after PANC-1 cells exposure to Y_2_O_3_-NPs. These findings are consistently with the fact that p53 upregulation leads to concurrent marked activation and overexpression of the mitochondrial apoptosis-inducer ND3 gene, and significant inactivation and downregulation of the anti-apoptotic Bcl2 gene expression^37,38^.

## Conclusion

In conclusion, the findings from this study highlight the promising potential of Y_2_O_3_-NPs as a valuable addition to current therapeutic approaches for pancreatic cancer. These nanoparticles demonstrated safety on normal HSF cells while exhibited a selective strong cytotoxicity towards PANC-1 cancer cells. This potent cytotoxicity may result from Y_2_O_3_-NPs induced excessive ROS generation, which damages DNA, disrupts the permeability of mitochondrial membranes, and dysregulates the expression of apoptotic, anti-apoptotic and mitochondrial genes, ultimately inducing apoptosis. This discovery offers a promising approach for enhancing pancreatic cancer treatment. Future clinical trials are essential to fully explore the safety and therapeutic efficacy of Y_2_O_3_-NPs in treating this aggressive disease. In vivo studies using animal models will provide a better understanding of their pharmacokinetics, bio-distribution, and potential off-target effects. These trials should also focus on determining the optimal dosage, assessing long-term effects, and ensuring the therapeutic potential of Y_2_O_3_-NPs without causing significant harm to healthy tissues. Additionally, combining Y_2_O_3_-NPs with other therapies may further enhance their effectiveness. Therefore, additional research, including in vivo studies and clinical trials, are recommended to fully assess the safety and clinical applicability of Y_2_O_3_-NPs for treating this pancreatic cancer disease.

## Data Availability

The datasets used and/or analyzed during the current study are available from the corresponding author on reasonable request.
